# ZiBuPiYin Recipe Protects db/db Mice from Diabetes-Associated Cognitive Decline through Improving Multiple Pathological Changes

**DOI:** 10.1371/journal.pone.0091680

**Published:** 2014-03-10

**Authors:** Jing Chen, Lina Liang, Libin Zhan, Yan Zhou, Luping Zheng, Xiaoxin Sun, Jin Gong, Hua Sui, Rujiao Jiang, Fuliang Zhang, Lin Zhang

**Affiliations:** 1 The Second Affiliated Hospital of Dalian Medical University, Dalian, Liaoning, China; 2 College (Institute) of Integrative Medicine, Dalian Medical University, Dalian, Liaoning, China; 3 Anatomy department of Dalian Medical University, Dalian, Liaoning, China; 4 Public Health department of Dalian Medical University, Dalian, Liaoning, China; National Institute of Health, United States of America

## Abstract

Multiple organ systems, including the brain, which undergoes changes that may increase the risk of cognitive decline, are adversely affected by diabetes mellitus (DM). Here, we demonstrate that type 2 diabetes mellitus (T2DM) db/db mice exhibited hippocampus-dependent memory impairment, which might associate with a reduction in dendritic spine density in the pyramidal neurons of brain, Aβ_1-42_ deposition in the prefrontal cortex (PFC) and hippocampus, and a decreased expression of neurostructural proteins including microtubule-associated protein (MAP2), a marker of dendrites, and postsynaptic density 95 (PSD95), a marker of excitatory synapses. To investigate the effects of the ZiBuPiYin recipe (ZBPYR), a traditional Chinese medicine recipe, on diabetes-related cognitive decline (DACD), db/db mice received daily administration of ZBPYR over an experimental period of 6 weeks. We then confirmed that ZBPYR rescued learning and memory performance impairments, reversed dendritic spine loss, reduced Aβ_1-42_ deposition and restored the expression levels of MAP2 and PSD95. The present study also revealed that ZBPYR strengthened brain leptin and insulin signaling and inhibited GSK3β overactivity, which may be the potential mechanism or underlying targets of ZBPYR. These findings conclude that ZBPYR prevents DACD, most likely by improving dendritic spine density and attenuating brain leptin and insulin signaling pathway injury. Our findings provide further evidence for the effects of ZBPYR on DACD.

## Introduction

Diabetes-associated cognitive decline (DACD) is one of central nervous systems (CNS) complications induced by diabetes mellitus (DM) [1], and has been recognized in humans [2], [3] and animal models [4], [5]. Thus, identification of treatment strategies for DACD has been an important research goal. More and more complementary and alternative therapies were used to facilitate the conventional treatment of diseases. Among these complementary and alternative therapies, traditional Chinese medicine is a popular component. Traditional Chinese medicine has the advantage of providing multiple therapeutic effects on multiple targets as compared with Western medicine, which usually focuses on a single target [6], and is now attracting more and more attention around the world [7]. However, efficacy and action mechanism of many traditional Chinese medicine have not yet been well understood. In this study, we examined the effects and action mechanisms of ZiBuPiYin recipe (ZBPYR) on DACD.

ZBPYR is derived from a modification of the Zicheng Decoction, which is a traditional Chinese medicine recipe recorded in the book of Bujuji by Cheng Wu during the Qing dynasty, and is used for clinical treatment of memory loss. Previous studies in our laboratory have demonstrated that ZBPYR improved learning and memory ability in DM rats induced by a high-fat diet combined with Streptozotocin (STZ) [8]. We also have revealed that the serum containing ZBPYR protected hippocampal neurons against amyloid β-peptide (Aβ) and glutamate induced neurotoxicity [9], [10].

In this study, we used db/db mouse as a DACD animal model, since it is reported that db/db mouse, an animal model of type 2 diabetes mellitus (T2DM), exhibits not only obesity, hyperglycemia, hyperinsulinemia, leptin and insulin resistance but also impaired hippocampus-dependent cognitive performance [5]. The aims of the present study were to (i) determine whether ZBPYR protects diabetic mice from DACD and (ii) explore the underlying targets or the action mechanisms of ZBPYR. Our results suggest that ZBPYR exhibited a significant activity in enhancing hippocampus-dependent memory in db/db mice. This activity may be related to the improvement of dendritic spine density, Aβ_1-42_ deposition and brain leptin and insulin signaling, also the inhibition of GSK3β overactivity by ZBPYR.

## Materials and Methods

### Ethics statement

All animal experiments were conducted in accordance with the NIH Principles of Laboratory Animal Care and the institutional guidelines for the care and use of laboratory animals at Dalian Medical University. All experiments were approved by the Committee on the Ethics of Animal Experiments of Dalian Medical University (Permit Number: SYXK (Liao) 2008**–**0002). All surgery was performed under anesthesia with ether (the usage of ether was approved by the Committee on the Ethics of Animal Experiments of Dalian Medical University), and all efforts were made to minimize suffering.

### Animals

Male 6- to 8-week-old C57BLKS/J-db/db mice and their age-matched non-diabetic littermates db/m mice were purchased from Nanjing Qingzilan Technology Co., Ltd. (Nanjing, Jiangsu Province, China) and housed in the specific pathogen-free (SPF) animal experiment center at Dalian Medical University. The animals were fed food and water ad libitum and housed at 24°C±2°C with 65%±5% humidity on a 12-h light/dark cycle. After 1 week acclimatization, db/db mice were randomly divided into 2 groups: a diabetes group (DM) and a diabetic mice treated with ZBPYR group (DM/ZBPYR).

### Preparation and administration of ZBPYR

The ZBPYR was composed of 12 crude herbs: Red Ginseng (Radix Ginseng Rubra), Common Yam Rhizome (Rhizoma Dioscoreae Oppositae), Indian Buead (poria), White Peony Root (Radix Paeoniae Alba), Dan shen Root (Radix Salviae Miltiorrhizae), White Hyacinth Bean (Semen Lablab Album), Lotus Seed (Semen Nelumbinis), Grassleaf Sweetflag Rhizome (Rhizoma Acori Tatarinowii), Thinleaf Milkwort Root (Radix Palygalae), Sandalwood (Lignum Santali Albi), Tangerine Red Epicarp (Exocarpium Citri Rubrum) and Liquorice Root (Radix Glycyrrhizae). All herbs were purchased from Dalian Metro Pharmaceutical Co., Ltd. (Dalian, Liaoning Province, China). The mixtures were soaked in 8 volumes (v/w) of distilled water for 30 min and then boiled for 90 min. The decoction was then concentrated to a final density of 3.29 g/ml and stored at 4°C. During a period of 6 weeks, DM/ZBPYR mice were orally administered ZBPYR at a dose of 0.1 ml/10 g body weight, while DM and control mice were orally administered an identical dose of ultrapure water (Milli-Q Integral Water Purification System, Millipore Corporation, Billerica, MA, USA).

### Random blood glucose and fasting serum insulin

Random blood glucose (RBG) was measured weekly to verify the development of diabetes in the db/db mice. Glucose levels in tail blood samples were determined using a glucometer (Roche, Mannheim, Germany). At the end of the administration period, mice were starved for 12 h, anesthetized with ether and blood samples collected from the inner canthus vein of the eye. Blood samples were then centrifuged at 3000 rpm for 15 min at 4°C for the separation of serum. The serum samples were frozen at −80°C until required for fasting serum insulin (FSI) measurement. FSI levels were assayed using an insulin radioimmunoassay kit (Atom High-tech, Beijing, China).

### Oral glucose tolerance test and insulin tolerance test

For oral glucose tolerance test (OGTT), mice were fasted for 14 h and administered a 50% (wt/wt) glucose solution (2 g/kg body weight) orally. Tail blood samples were then collected at 0, 30, 60 and 120 min after the glucose administration. For insulin tolerance test (ITT), mice were fasted for 6 h, injected intraperitoneally with regular human insulin (0.75 U/kg body weight; Novolin, Novo Nordisk (China) Pharmaceutical Co., Ltd., Tianjin, China), and glucose levels monitored at 0, 15, 30, 60, 90, and 120 min after insulin injection. All glucose levels were determined using a glucometer (Roche, Mannheim, Germany).

### Morris water maze test

Taking into account light conditions might be an important factor in the behavior test, and mice are more active in dark phase, so the light/dark cycle was reversed during the Morris water maze test. The test was performed in the morning under conditions of low noise and dim light [11], [12]. The light intensity was set up according to the paper of Verónica S. Valentinuzzi et al [13], provided by indirect three incandescent lamps (100 W) connected to a dimmer, was 11 to 24 lux at the water surface. Briefly, the test was undertaken in a circular pool (diameter 100 cm, height 50 cm, Institute of Materia Medica, Chinese Academy of Medical Sciences, Beijing, China), filled with water made opaque with milk power and maintained at 26°C±1°C. The experiment was performed daily for 6 days. On the 1st day, mice were permitted to swim freely in the pool for 120 s without the platform (diameter 9 cm, height 29 cm) to adapt to the new conditions. Over the following 4 days, mice underwent 4 trials per day at intervals of 60 s. On these occasions, the platform location was submerged 1 cm below the water surface and was fixed, while the starting points were changed. Each trial lasted until the animal found the platform or for a maximum observation period of 120 s. Animals that failed to find the platform within the maximum observation period were guided to the platform by the observers. On the day after the last acquisition training session, animals were tested in a single 120 s probe test without the platform. During this period, three parameters were recorded via an automatic photographic recording and analysis system (EthoVision, Noldus Information Technology b.v., Wageningen, The Netherlands): (1) the time to reach the platform (escape latency); (2) the swimming time in the target quadrant where the platform had been located during training; and (3) the number of times the animal crossed the site from which the original platform had been removed. On the 6th day, a visible-platform test was undertaken, where the platform was located 1 cm over the water surface and placed at a position different from the previous test. During this test, the experimental procedures were exactly the same as previous tests, and the escape latency and swimming distance were recorded.

### Sample preparation

For western blotting, the animals were anesthetized with ether and decapitated. Cerebral cortex and hippocampus was rapidly dissected via surgery on ice. All samples were immediately frozen in liquid nitrogen and stored at −80°C until required. Cerebral cortex and hippocampus samples were homogenized in ice-cold lysis buffer (0.125 M Tris HCl (pH 6.8),0.2 M DTT,4% SDS,20% Glycerol). The lysates were sonicated for 10 min and centrifuged at 15,000×g for 5 min to remove insoluble debris. Protein concentrations in the supernatants were determined using a Minim Spectrophotometer (NanoVue™ Plus, GE Healthcare, Amersham Place, Little Chalfont, Buckinghamshire, HP79NA, UK).

For immunohistochemistry staining, the animals were deeply anesthetized with ether and sacrificed by intracardiac injection of 0.9% saline solution followed by fixative (4% paraformaldehyde in 0.1 M phosphate buffer, PB, pH 7.4). Brains were removed and post-fixed in the same fixative overnight at 4°C and then in a 30% sucrose solution (in PB) at 4°C until they floated. Coronal sections (8 µm) from the PFC and hippocampus were cut using a frozen microtome (Leica, Wetzlar, Germany). Sections were collected on clean Poly-L-lysine coated microscope slides (3 sections/slide), and the sections from different group animals were on the same slide to reduce the effects of experimental manipulations and conditions on the results. All slides were stored at −20°C until required.

### Western blotting

Protein (50 µg per sample) was separated on 8%**–**15% Tris-glycine polyacrylamide gels and transferred to nitrocellulose membranes. Immunoblots were blocked for 1 h in Tris-buffered saline Tween-20 (TBST, 20 mM Tris-HCl, 150 mM NaCl, pH 7.5, 0.05% Tween 20) containing 5% skim milk. The blots were then incubated with primary antibodies in TBST at 4°C overnight. Membranes were washed three times with TBST and then incubated with secondary antibody for 2 h at room temperature. Membranes were washed again and developed with Enhanced Chemiluminescence (ECL). Primary antibodies used from Cell Signaling Technologies (Danvers, MA, USA) were: suppressor of cytokine signaling 3 (SOCS3) (#2932, 1∶800), Janus kinase 2 (JAK2) (#3230, 1∶800), p-Tyr^1007/1008^JAK2 (#3776, 1∶800), protein kinase B (Akt) (#9272, 1∶800), p-Ser^473^Akt (#4058, 1∶800), GSK3α (#4337,1∶800), p-Ser^21^GSK3α (#9316, 1∶800), GSK3β (#9315, 1∶800), p-Ser^9^GSK3β (#9322, 1∶800), PSD95 (#3409, 1∶800), MAP2 (#4542, 1∶800), p-Thr^1620/1623^MAP2 (#4544, 1∶800). Primary antibody directed against insulin receptor substrate 2 (IRS2) (Millipore Corporation, Billerica, MA, USA, #MABS15, 1∶2000), p-Ser^731^IRS-2 (Abcam plc, Cambridge, UK, #ab3690, 1∶200) and β-actin (Sigma-Aldrich, St Louis, MO, USA, #A2228, 1∶2000) were also used in the experiments. Goat anti-rabbit (#NA9340, 1∶2000) or anti-mouse (#NA9310, 1∶2000) (GE Healthcare, Buckinghamshire, UK) were used as secondary antibodies.

### Immunohistochemistry staining and quantitative analysis

Immunohistochemistry staining was carried out using a Streptavidin/Peroxidase staining kit (SP9001, Beijing Zhongshan Golden Bridge Biotechnology Co., Ltd., Beijing, China) according to the manufacturer's instructions. Briefly, after incubation at room temperature for 30 min, sections were washed three times for 10 min in 0.01 M phosphate-buffered sodium (PBS, pH 7.4) and blocked with 3% hydrogen peroxide for 10 min at room temperature to quench endogenous peroxidases. The sections were then incubated with solution A (Normal Goat Serum), and then incubated with primary antibodies at 4°C overnight. The following primary antibodies were used: MAP2 (Sigma-Aldrich, St Louis, MO, USA, #M3696, 1∶100), Aβ_1-42_ (Abcam plc, Cambridge, UK, #ab10148, 1∶100) and PSD95 (Cell Signaling Technologies, Danvers, MA, USA, #3409, 1∶200). In order to exclude false-positive results, we established a negative control group that used PBS instead of primary antibody. The sections were then further processed using solution B (biotinylated goat anti-rabbit IgG) and solution C (horseradish peroxidase-labeled streptavidin working solution). Immunoreactivity was developed using diaminobenzidine (DAB, ZLI-9018, Beijing Zhongshan Golden Bridge Biotechnology Co., Ltd., Beijing, China) until suitable staining had developed. The sections were then counter-stained with hematoxylin, dehydrated through graded alcohol and rinsed in xylene before cover-slipping with balsam resinous medium.

The images from CA1, CA3 and the DG regions of the hippocampus and prefrontal cortex (PFC) were captured using an upright microscope (Leica, Wetzlar, Germany). The quantitative analysis was carried out using the “measure integrated optical density” (IOD) function of Image Pro-Plus version 6.0 and average IOD was used for statistical analysis.

### Golgi staining and analysis of dendritic spines density

For Golgi staining, fixed brain tissue was treated with a 3.5% solution of potassium dichromate for 4**–**5 days, followed by 10 days in a solution of 1% silver nitrate. The sections were then washed with distilled water, dehydrated and cleared in successive baths of 95% (1**–**2 h) and 100% (2 h) alcohol followed by 1 h in an ether anhydrous alcohol mixture (1∶1). After embedding with collodion (2%**–**4% 1 h, 8% 1 h), coronal sections (200 µm) from the PFC and hippocampus were obtained using a sliding slicer. These sections were collected onto clean 2% gelatin-coated glass microscope slides and cover-slipped with balsam resinous medium.

For analysis of dendritic spine density, images were captured using an upright microscope (Leica, Wetzlar, Germany) and processed qualitatively with Image Pro-Plus version 6.0. The apical spines on secondary and tertiary dendrites of hippocampal CA1 and CA3 pyramidal neurons, granule cells of the DG and layer II/III pyramidal neurons in the PFC were counted per 50 µm of dendritic segment length. For analysis of dendritic spine density in different brain regions, we chose three mice from each group, and five brain sections from each mouse, and five neurons from each brain section, so totally 25 neurons were analyzed for each mouse in the hippocampus or prefrontal cortex. Only spines from neurons that were adequately stained and whose branches were not obscured by other dendrites, blood vessels, or non-descript precipitates were quantified.

### Statistical analysis

Statistical analysis was performed using either an ANOVA (equal variance) or a Welch's ANOVA (unequal variance) test. Data from the Morris water maze test was analyzed using a repeated-measures analysis of variance for comparisons among trials, while an unpaired Student's t test was used for comparisons among different groups in a given block and for the comparison of other results. The difference was considered to be statistically significant when *p*≤0.05.

## Results

### ZBPYR improves spatial learning and memory performance

**Figure 1:**
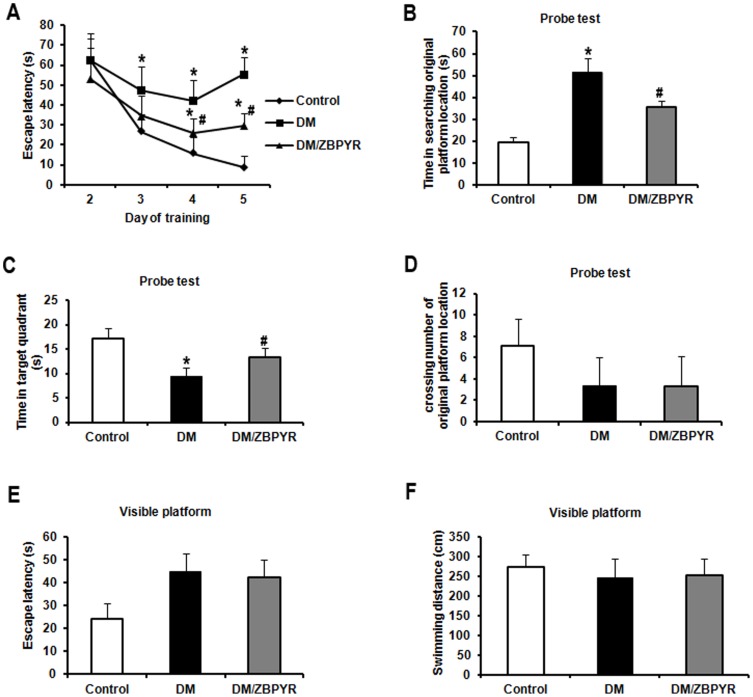
Effects of ZBPYR on the Morris water maze test in db/db mice. **(A)** Learning performance of the animals was analyzed in the training trials by escape latency. DM/ZBPYR mice had shorter escape latency on the 4th and 5th day of training. (**B–D**) Memory retrieval performance was investigated in the probe test as the time required to search for the original platform location (**B**), time in the target quadrant where the platform had been located during training trials (**C**), and the number of crossing over the original platform location (**D**). (**E–F**) Performance in the visible platform version of the Morris water maze, which is not hippocampus-dependent. Escape latency (**E**) and swimming distance (**F**) were analyzed. Values are means ± S.D. from 17 mice in each group. *p<0.05 compared to control; ^#^ *p*<0.05 compared to DM.

As shown in Figure 1A, the escape latency of DM mice was significantly longer than that of control mice from 3rd day to 5th day (3rd day 1.77 times, 4th day 2.68 times, 5th day 6.39 times). When compared to DM mice, DM/ZBPRY mice exhibited shorter escape latency on the 4th (0.62 times) and 5th day (0.54 times) of the training trials.

In the probe test, we observed that DM/ZBPRY mice required less time (0.68 times) to locate the original platform position than DM mice (Figure 1B). The swimming time of DM mice in the target quadrant where the platform had been located during training tests was significantly shorter (0.70 times) than that of DM/ZBPYR mice (Figure 1C). There was no significant difference among the groups in the number of times the mice crossed the original platform location (Figure 1D).

Performance in the visible platform version of the Morris water maze test, which is not hippocampus-dependent [14], [12], was similar among all groups in terms of escape latency and swimming distance (Figure 1E and F).

### ZBPYR increases dendritic spines density

**Figure 2:**
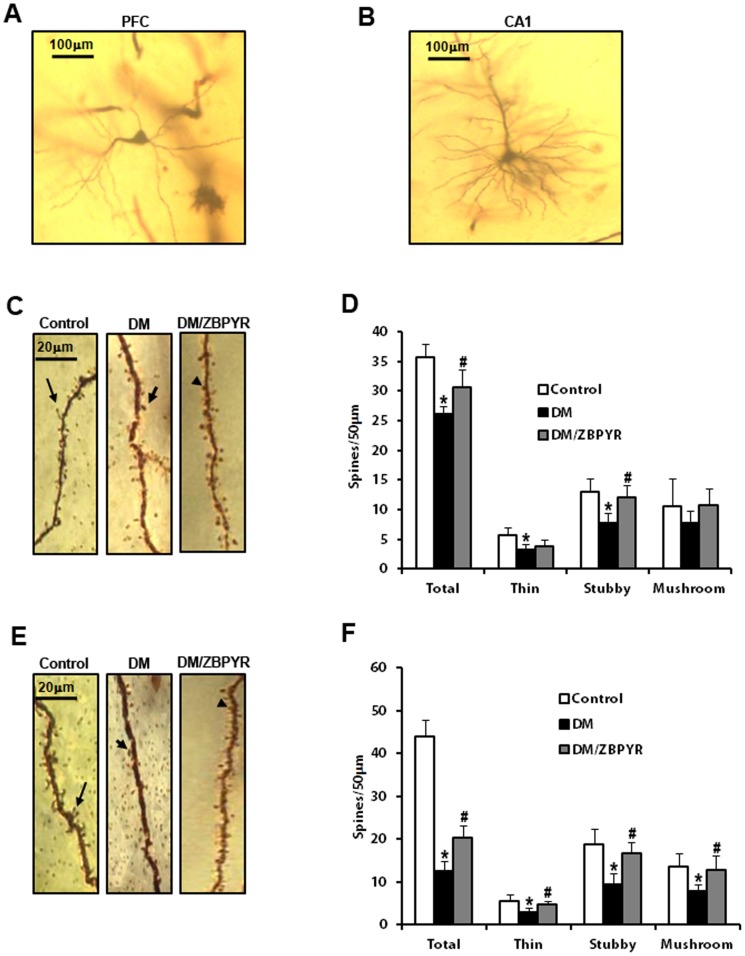
Effects of ZBPYR on dendritic spines in different regions of brain. (**A–B**) Representative examples of a PFC neuron (**A**) and a CA1 neuron (**B**) visualized with Golgi staining. Scale bar = 100 µm. (**C**) Representative images of Golgi-stained PFC neurons from the different groups. Long arrow indicates a thin spine, short arrow indicates a mushroom spine and triangle indicates a stubby spine. Scale bar = 20 µm. (**D**) ZBPYR significantly increased total dendritic spine density and stubby spine quantity over a dendritic segment length of 50 µm. (**E**) Representative images of Golgi-stained hippocampal CA1 neurons from the different groups are shown. Long arrow indicates a thin spine, short arrow indicates a mushroom spine and triangle indicates a stubby spine. Scale bar = 20 µm. (**F**) ZBPYR increased total dendritic spine density and all three types spines quantity over a dendritic segment length of 50 µm. Values are means ± S.D. from 3 mice in each group. *p<0.05 compared to control; ^#^ *p*<0.05 compared to DM.

Since the changes in dendritic spines may be a major cause of learning and memory impairment [15], [16], [17], we analyzed dendritic spine density in Golgi-stained neurons in the hippocampus and prefrontal cortex (PFC). In our study, Golgi staining was observed in the dendritic shafts and spines on secondary and tertiary dendrites of pyramidal neurons from layer II/III of the PFC (Figure 2A) and hippocampus (Figure 2B).

Analysis of the dendritic spine density in the PFC revealed that there was a significant difference among groups. When compared with the control group, the total number of dendritic spines along a 50-µm dendritic segment was significantly smaller in DM mice (0.29 times), while ZBPYR treatment mitigated these changes (1.64 times) (Figure 2C and D). Moreover, we also observed fewer dendritic spines on hippocampal CA1 pyramidal neurons in DM mice (0.73 times) than control mice, while ZBPYR treatment increased the density of dendritic spines in this area (1.18 times) (Figure 2E and F).

In addition, density of different types of spines observed over a 50-µm segment of the dendrite was also analyzed. Dendritic spines were divided into 3 types: 1) thin-type, a long, narrow protrusion terminating in a small bulbous head; 2) stubby-type, small protrusions lacking a clearly distinguishable neck and head portion, and 3) mushroom-type, well defined neck and very voluminous head. Thin- and stubby-type spines on pyramidal neurons in the PFC of control mice were observed in greater numbers (Thin: 1.89 times, stubby: 1.98 times) than that of DM mice. Furthermore, ZBPYR significantly increased stubby-type spines (1.77 times) in DM/ZBPYR mice (Figure 2D). The density of three different types of spines in the hippocampal CA1 pyramidal neurons of DM mice were fewer (Thin: 0.58 times, stubby: 0.60 times, mushroom: 0.73 times) than those in control mice. Moreover, it is interesting that ZBPYR treatment could increase all three different types of spines in hippocampal CA1 (Thin: 1.47 times, stubby: 1.54 times, mushroom: 1.39 times) (Figure 2F).

We also observed dendritic spine density and density of different types of spines on hippocampal CA3 pyramidal neurons and granule cells of the dentate gyrus (DG), but we did not observe the effects of ZBPYR in these regions (Figure S1).

### ZBPYR reduces Aβ_1-42_ deposition and increases the expressions of MAP2 and PSD95

**Figure 3:**
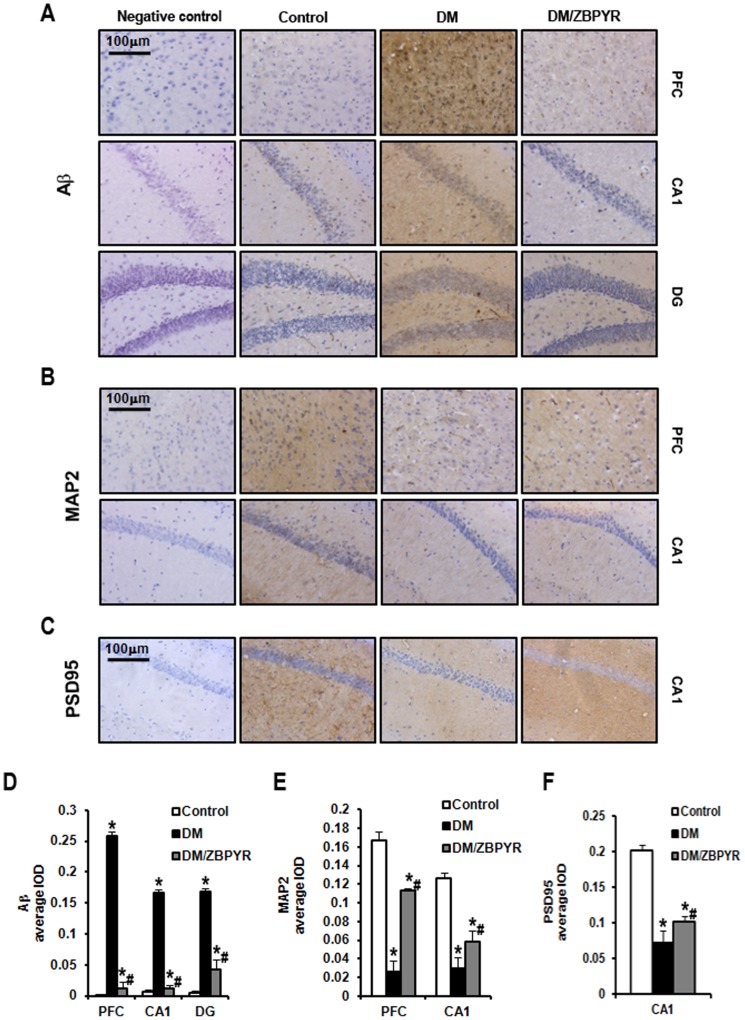
Effects of ZBPYR on Aβ_1-42_ deposition and the expression of neurostructural proteins in different regions of brain. (**A**) Aβ_1-42_ deposition in the PFC, hippocampal CA1 and DG were visualized using immunohistochemistry. (**B**) The expression of MAP2 in the PFC and hippocampal CA1 was visualized using immunohistochemistry. (**C**) The expression of PSD95 in the hippocampal CA1 was visualized using immunohistochemistry. (**D–F**) The average IOD of the immunoreactive area for Aβ_1-42_, MAP2 and PSD95, are presented as bar graphs. ZBPYR significantly reduced Aβ_1-42_ deposition in the PFC and hippocampal CA1 and DG (**D**). ZBPYR significantly increased the expression of MAP2 in the PFC and hippocampal CA1 (**E**). ZBPYR significantly increased the expression of PSD95 in the hippocampal CA1 (**F**). Scale bar = 100 µm. Values are means ± S.D. from 3 mice in each group. *p<0.05 compared to control; ^#^ *p*<0.05 compared to DM.

The pathology of DACD is similar to that of Alzheimer's Disease (AD), which is characterized by the presence of senile plaques composed of aggregated extracellular Aβ protein. Thus, in addition to changes in dendritic spines, we hypothesized that Aβ deposition would be observed in the DACD brain, and that ZBPYR would be involved in the process of Aβ deposition. In order to exclude false-positive results in these experiments, we established a negative control group. As shown in Figure 3A and D, almost no Aβ_1-42_ immunoreactivity was observed in the PFC and hippocampal CA1 and DG in control mice. When compared with control mice, Aβ_1-42_ immunoreactivity was much more intense in DM mice and ZBPYR treatment resulted in a decrease in the degree of staining. We did not, however, observe any significant changes in the hippocampal CA3 after ZBPYR treatment (Figure S2).

MAP2 is often utilized as a marker for the dendritic cytoskeleton [18]. In our study, we identified a significant reduction in MAP2 immunoreactivity in the PFC (0.16 times) and hippocampal CA1 (0.24 times) of DM mice when compared to the control mice. And we found ZBPYR treatment obviously increased MAP2 immunostaining in these two brain areas (PFC: 4.28 times, CA1: 1.93 times) (Figure 3B and E). However, MAP2 expression was not statistically altered in the CA3 and DG of the hippocampus when comparing DM and DM/ZBPYR mice (Figure S2).

The distal tip of the dendritic spine head contains a postsynaptic density (PSD) region, which is an electron-dense structure specialized for postsynaptic signaling and plasticity, and consists of glutamate receptors, signaling molecules and scaffolding proteins [19]. As PSD95 is a key scaffolding protein located in the PSD, we measured PSD95 expression using immunohistochemistry staining. The results revealed significant differences in PSD95 expression between DM and control mice in the hippocampal CA1 (0.36 times) (Figure 3C and F), in the PFC (0.40 times) and CA3 (0.53 times), and the DG (0.40 times) of the hippocampus (Figure S2). Interestingly, similar to the results for MAP2 expression, the CA1 region showed an obvious increase (1.42 times) in PSD95 expression after ZBPYR administration (Figure 3C and F), while PSD95 expression in the PFC and other areas of the hippocampus was not statistically altered (Figure S2).

### ZBPYR affects brain leptin and insulin signaling

Due to the expression of a mutant leptin receptor, db/db mice show severe leptin resistance and insulin resistance. To assess brain leptin and insulin signaling in our model, we examined the expression of SOCS3, which is known to be a major negative regulator of leptin and insulin signaling [20], [21], JAK2 and p-Tyr^1007/1008^JAK2, IRS2 and p-Ser^731^IRS2, Akt and p-Ser^473^Akt in the hippocampus and cerebral cortex using western blotting.

**Figure 4:**
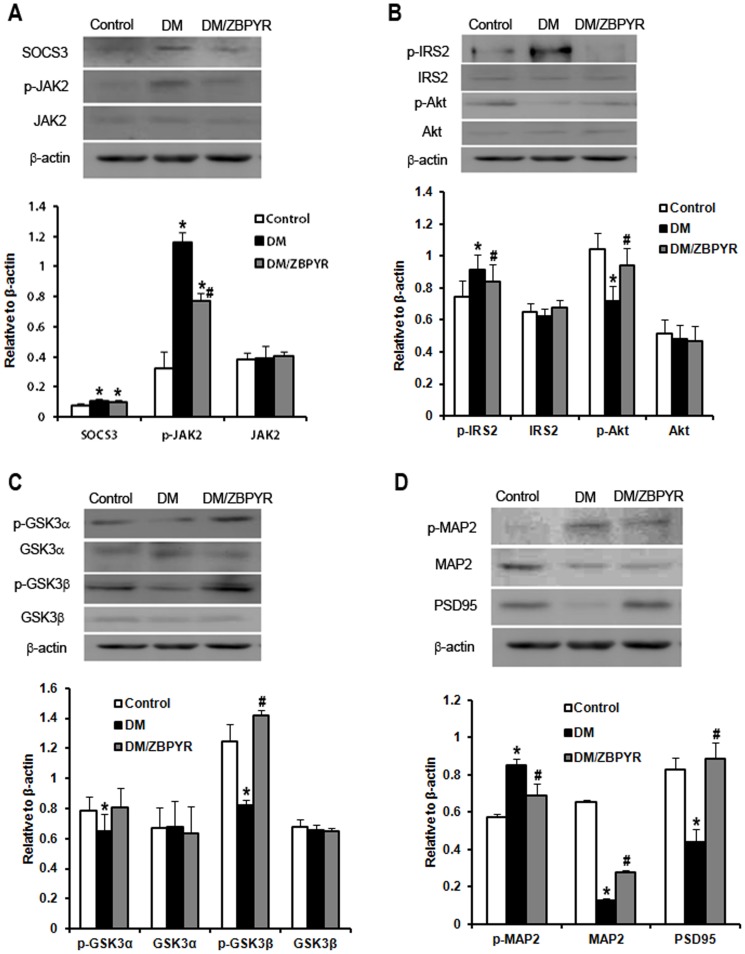
Effects of ZBPYR on brain leptin and insulin signaling. GSK3 activity and neurostructural protein expression in the hippocampus. (**A**) ZBPYR did not alter SOCS3 expression, but significantly decreased p-JAK2 (Tyr^1007/1008^) expression. (**B**) ZBPYR reduced IRS2 that is phosphorylated at serine 731 and increased Akt phosphorylated at serine 473. (**C**) ZBPYR significantly impaired GSK3β activity by increasing p-Ser^9^GSK3β expression. (**D**) ZBPYR significantly increased MAP2 and PSD95 expression, and reduced MAP2 that is phosphorylated on tyrosine 1620/1623. Representative Western blots and bar graphs of gray-scale analysis are shown. β-actin was used as a loading control. Values are means ± S.D. from 4 mice in each group. *p<0.05 compared to control; ^#^ *p*<0.05 compared to DM.

**Figure 5:**
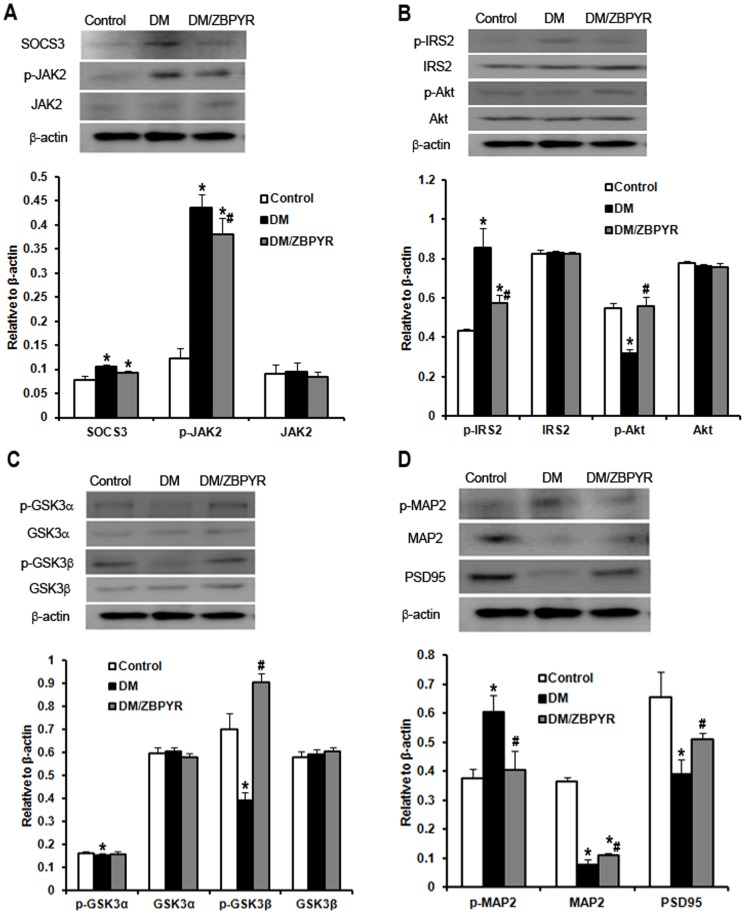
Effects of ZBPYR on brain leptin and insulin signaling. GSK3 activity and neurostructural protein expression in the cerebral cortex. (**A**) ZBPYR did not alter SOCS3 expression, but significantly decreased p-JAK2 (Tyr^1007/1008^) expression. (**B**) ZBPYR reduced p-IRS2 at serine 731 and increased p-Akt at serine 473. (**C**) ZBPYR significantly impaired GSK3β activity by increasing p-Ser^9^GSK3β expression. (**D**) ZBPYR significantly increased MAP2 and PSD95 expression, and reduced MAP2 that is phosphorylated on tyrosine 1620/1623. Representative Western blots and bar graphs of gray-scale analysis are shown. β-actin was used as a loading control. Values are means ± S.D. from 4 mice in each group. *p<0.05 compared to control; ^#^ *p*<0.05 compared to DM.

SOCS3 expression was increased in the hippocampus (Figure 4A) and cerebral cortex (Figure 5A) of DM and DM/ZBPYR mice when compared to control mice, while no differences were detected between DM and DM/ZBPYR mice. Next, the results identified that total JAK2 expression was not significantly changed in the different brain tissues among the three groups (Figure 4A and 5A). When compared with control mice, p-Tyr^1007/1008^JAK2 expression was increased significantly in the hippocampus (Figure 4A) and cerebral cortex (Figure 5A) of DM and DM/ZBPYR mice. Moreover, ZBPYR was able to reduce JAK2 hyper-phosphorylation (Figure 4A and 5A).

Our results revealed no changes in the total levels of IRS2 and Akt in either brain region among the three groups (Figure 4B and 5B). However, we did observe an apparent reduction in levels of p-Ser^473^Akt and a significantly increased p-Ser^731^IRS2 in the hippocampus (Figure 4B) and cerebral cortex (Figure 5B) of DM mice when compared to control mice, and that ZBPYR showed an ability to ameliorate these alterations (Figure 4B and 5B).

### ZBPYR inhibits GSK3 overactivity and increases the expressions of dendritic cytoskeleton proteins

As impaired insulin signaling is known to affect GSK3 activity [22], we next investigated whether GSK3 activity was altered in our model. We observed a reduction in phosphorylated GSK3α at Ser21 and phosphorylated GSK3β at Ser9 in the hippocampus (Figure 4C) and cerebral cortex (Figure 5C) of DM mice, illustrating that GSK3 activity was increased. After ZBPYR administration, p-Ser^9^GSK3β expression was enhanced in the hippocampus and cerebral cortex of the DM/ZBPYR mice (Figure 4C and 5C). We did not detect changes in the levels of total GSK3α and GSK3β.

Phosphorylation of MAP2 induced by GSK3 modulates its association with microtubules and regulates microtubule stability [23]. In our study, we found that DM mice showed a decrease in MAP2 protein expression in the hippocampus (Figure 4D) and cerebral cortex (Figure 5D) when compared to control mice. In addition, DM mice appeared to have elevated levels of phosphorylated MAP2 in the hippocampus (Figure 4D) and cerebral cortex (Figure 5D). Treatment with ZBPYR appeared to ameliorate these alterations (Figure 4D and 5D). The results also showed that PSD95 expression in the hippocampus (Figure 4D) and cerebral cortex (Figure 5D) were sharply decreased in DM mice when compared with control mice. Moreover, these reductions were restored following treatment with ZBPYR (Figure 4D and 5D).

### Effects of ZBPYR on peripheral glucose homeostasis and insulin sensitivity

**Figure 6:**
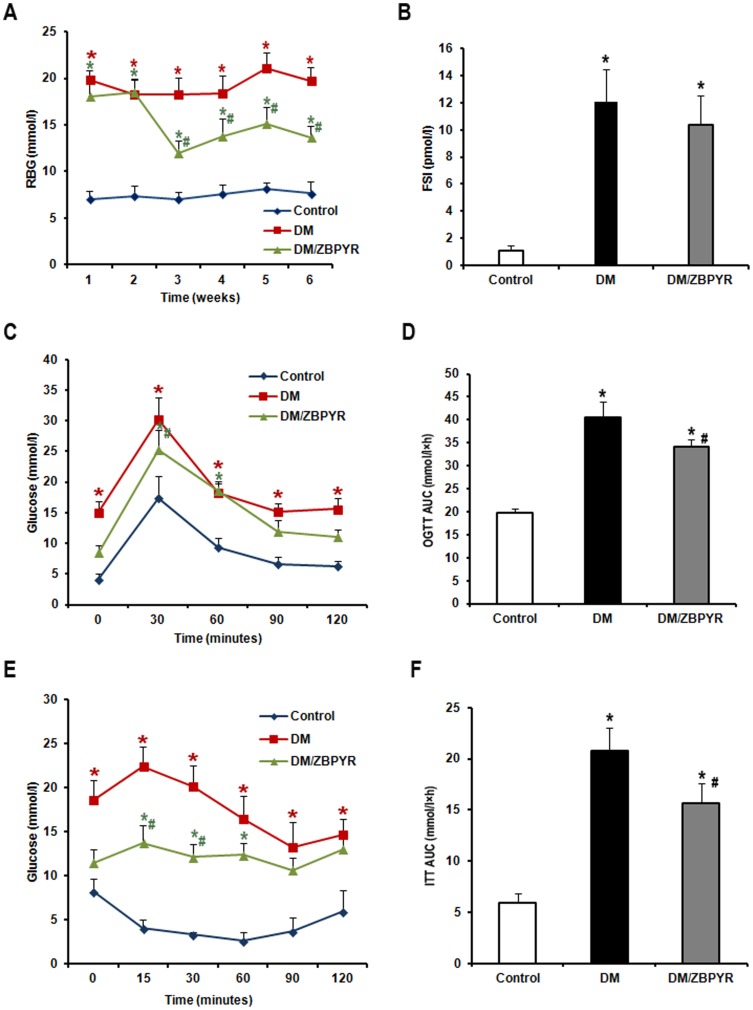
The anti-diabetic effects of ZBPYR on db/db mice. (**A**) RBG was examined weekly during ZBPYR administration. ZBPYR significantly reduced glucose levels from the 3rd week to the 6th week. (**B**) FSI was accessed at the end of the administration period and after the animals had been starved for 12 h. (**C–D**) OGTT (2 g/kg body weight) was performed at the end of the administration period and after the animals had been starved for 14 h. Actual glucose levels were measured at the indicated times (**C**), and then the AUC was analyzed (**D**). (**E–F**) ITT (0.75 U/kg body weight) was performed at the end of the administration period and after the animals had been starved for 6 h. Actual glucose levels were measured at the indicated times (**E**), and then the AUC was analyzed (**F**). Values are means ± S.D. from 17 mice in each group. *p<0.05 compared to control; ^#^ *p*<0.05 compared to DM.

The results revealed that RBG levels in DM/ZBPYR mice were significantly reduced (3rd: 0.65 times, 4th: 0.75 times, 5th: 0.72 times, 6th: 0.69 times) between the 3rd and 6th weeks of treatment when compared to DM mice (Figure 6A). There was no significant difference in FSI levels when comparing DM/ZBPYR mice and DM mice at the end of the treatment period (Figure 6B). As shown in Figure 6C, in the OGTT, when compared to DM mice, ZBPYR administration was found to reduce blood glucose levels, especially at 30 min after a glucose load (0.83 times). The ITT results revealed that ZBPYR also significantly improved the glucose response to an insulin challenge at 15 min (0.61 times) and 30 min (0.60 times) (Figure 6E). When the area under the curve (AUC) of OGTT (Figure 6D) and ITT (Figure 6F) was compared between groups, DM/ZBPYR mice showed a significant reduction (OGTT: 0.85 times, ITT: 0.76 times) when compared with DM mice.

## Discussion

Although the pathogenesis of DACD is still not well understood, pharmacological treatment for this disease warrants exploration. ZBPYR was initially created according to the traditional Chinese medical theory in order to improve memory ability. Therefore, this promoted an investigation of the protective effects of ZBPYR on DACD. In the present study, we found that ZBPYR was able to improve DACD in db/db mice.

The results of Morris water maze test demonstrate that db/db mice exhibited severe cognitive deficits, which are consistent with previous findings [5], [24], [25]. ZBPYR enhanced learning performance on the 4th and 5th day of the training test and memory retrieval performance in the probe test in DM/ZBPYR mice, suggesting that ZBPYR has a beneficial effect in the treatment of DACD. Our data suggest that ZBPYR might act this effect through increasing dendritic spine density and reducing Aβ deposition. Since previous studies have demonstrated that learning and memory processes depend on synapses [26] and dendritic spines are the major sites of excitatory synapse formation [27]. In this study, db/db mice exhibited decreased dendritic spine density in different brain region, and ZBPYR was efficacious for recovering dendritic spine density, moreover, much more effective in the PFC and hippocampal CA1. In addition, ZBPYR increased the density of stubby-type spines in the PFC and hippocampal CA1, suggesting that the neuroprotective function of ZBPYR on DACD may depend on increasing the density of stubby-type spines in several brain regions, thereby enhancing memory ability. Moreover, according to findings from ours and other laboratories, the PFC is also a noteworthy brain area for cognitive function and memory loss [28], [29]. It is well documented that Aβ deposition is associated with learning and memory ability [30], [31]. In current study, db/db mice exhibited Aβ_1-42_ deposition in several brain regions, and ZBPYR showed a significant reduction action on Aβ_1-42_ deposition in the PFC and CA1 and CA3 of hippocampus. Thus, our findings raise the possibility that the effect of ZBPYR on prevention of DACD is dependent of Aβ pathology.

In the molecular mechanism of ZBPYR action, the activity of ZBPYR may be associated with the enhancement of leptin and insulin signaling in the cerebral cortex and hippocampus, since there are several lines of evidence suggesting that leptin and insulin signaling play an important role in cognitive performances and learning and memory processes for humans [32], [33] and animals [34], [35]. This present study revealed that p-Tyr^1007/1008^JAK2 was up regulated in the cerebral cortex and hippocampus of db/db mice. ZBPYR ameliorated JAK2 hyper-phosphorylation in these regions, suggesting that ZBPYR may affect leptin signaling by regulating p-Tyr^1007/1008^JAK2. In the CNS, activated JAK2 leads to the activation of a series of downstream signaling pathways, including Akt [36], [37]. It is well established that Akt is a key marker protein of insulin signaling, and which mediates the effect of insulin via important intracellular signaling cascades including the IRS/PI3K/Akt pathway [38]. Moreover, recent epidemiological evidence suggests that CNS insulin resistance is a risk factor for cognitive decline [39]. In this study, we found that p-IRS2 was increased and p-Akt was decreased in the cerebral cortex and hippocampus of db/db mice, illustrating that central insulin signaling of db/db mice was impaired. Our study confirmed that ZBPYR could correct CNS leptin and insulin resistance by regulating p-JAK2, p-IRS2 and p-Akt.

In addition, ZBPYR exhibited a strong activity in the inhibition of GSK3. GSK3 is a downstream substrate of Akt and sensitive to glucose, since the phosphorylation states of GSK3 in both the cerebral cortex and hippocampus were found to be influenced by fluctuations in the peripheral blood glucose concentration [40]. It is reported that GSK3 involved in the process of Aβ deposition [41] and regulated developmental processes including synaptic plasticity [42]. In our study, we revealed that ZBPYR could prohibit excessive GSK3β activity, although it has no effect on GSK3α activity. GSK3β implicated in the regulation of microtubule dynamics by phosphorylating MAPs, including MAP2. Phosphorylation of MAP2 inhibited MAP2 from binding to microtubules and decreased microtubule stability [43]. In current study, we observed reduced expression of MAP2 and PSD95, enhanced expression of p-MAP2 in the cerebral cortex and hippocampus of db/db mice by western blotting. Moreover, immunohistochemical analysis revealed a decreased MAP2 and PSD95 immunoreactivity in the PFC and hippocampus of db/db mice. ZBPYR treatment appeared to oppose these pathology changes, especially selectively restored MAP2 expression in the PFC and hippocampal CA1 and selectively restored PSD95 expression in hippocampal CA1. Taken together, the present findings provide molecular biological evidence for the preventive effects of ZBPYR on DACD.

DM is associated with multiple adverse effects on the brain, some of which may result primarily from direct consequences of chronic hyperglycemia. Since herbal extracts have been reported to reduce blood glucose in animals [44], [45], we were interested in the anti-diabetic effect of ZBPYR. In the present study, we found that ZBPYR has a powerful activity in glucose homeostasis and insulin sensitivity. ZBPYR abrogated the elevation in RBG following 3 weeks of treatment. In addition, we identified that ZBPYR significantly improve peripheral glucose tolerance and insulin sensitivity, by comparing the AUC of OGTT and ITT, respectively. These results revealed that ZBPYR elicited anti-diabetic effects, which may be via the maintenance of peripheral glucose homeostasis and enhancing the efficacy of peripheral insulin.

In summary, this study concludes that ZBPYR is able to improve DACD in db/db mice. It is possible that the beneficial effects of ZBPYR on cognitive impairment may have been attributable to reversing preexisting pathological changes, including dendritic spine density and Aβ deposition. The data also suggest that the activity of ZBPYR may be related to improve brain leptin and insulin signaling and peripheral high glucose environment.

## Acknowledgments

We thank the Key Lab for Basic Research of Critical and Refractory Diseases with Integrated Traditional and Western Medicine, Liaoning Province, China.

## Supporting Information

Figure S1 **Effects of ZBPYR on dendritic spines in CA3 and DG of hippocampus.** **(A)** In the CA3 of hippocampus in DM mice, the total dendritic spine density, thin-type spine density and mushroom-type spine density were significantly decreased. **(B)** In the DG of hippocampus in DM mice, the total dendritic spine density and the density of thin- and stubby-type spine were significantly decreased. Values are means ± S.D. from 3 mice in each group. *p<0.05 compared to control.
(TIF)

Figure S2 **Effects of ZBPYR on Aβ_1-42_ deposition and the expression of neurostructural proteins in different brain region.** **(A)** Aβ_1-42_ deposition in the hippocampal CA3. **(B)** MAP2 expression in the hippocampal CA3 and DG. **(C)** The expression of PSD95 in the PFC, hippocampal CA3 and DG. **(D–F)** The average IOD of Aβ_1-42_ (**D**), MAP2 (**E**) and PSD95 (**F**) are shown as bar graphs. Values are means ± S.D. from 3 mice in each group. *p<0.05 compared to control.
(TIF)

## References

[1] Ahtiluoto S, Polvikoski T, Peltonen M, Solomon A, Tuomilehto J, et al (2010) Diabetes, Alzheimer disease, and vascular dementia: a population-based neuropathologic study. Neurology 75: 1195–1202. 20739645 10.1212/WNL.0b013e3181f4d7f8

[2] Profenno LA, Porsteinsson AP, Faraone SV (2010) Meta-analysis of Alzheimer's disease risk with obesity, diabetes, and related disorders. Biological Psychiatry 67: 505–512. 19358976 10.1016/j.biopsych.2009.02.013

[3] Kopf D, Frölich L (2009) Risk of incident Alzheimer's disease in diabetic patients: a systematic review of prospective trials. Journal of Alzheimer's Disease 16: 677–685. 10.3233/JAD-2009-1011 19387104

[4] Sharma AN, Elased KM, Garrett TL, Lucot JB (2010) Neurobehavioral deficits in db/db diabetic mice. Physiology & Behavior 101: 381–388. 20637218 10.1016/j.physbeh.2010.07.002 PMC3098504

[5] Zhao Q, Matsumoto K, Tsuneyama K, Tanaka K, Li F, et al (2011) Diabetes-Induced Central Cholinergic Neuronal Loss and Cognitive Deficit Are Attenuated by Tacrine and a Chinese Herbal Prescription Kangen-Karyu: Elucidation in Type 2 Diabetes db/db Mice. J Pharmacol Sci 117: 230–242. 22083044 10.1254/jphs.11115fp

[6] Piao YL, Liang XC (2012) Chinese Medicine in Diabetic Peripheral Neuropathy: Experimental Research on Nerve Repair and Regeneration. Evidence-Based Complementary and Alternative Medicine 2012: 1–13. 10.1155/2012/191632 PMC3426291 22927874

[7] Wen Z, Wang Z, Wang S, Ravula R, Yang L, et al (2011) Discovery of Molecular Mechanisms of Traditional Chinese Medicinal Formula Si-Wu-Tang Using Gene Expression Microarray and Connectivity Map. PLoS ONE 6: e18278. 21464939 10.1371/journal.pone.0018278 PMC3065471

[8] Shi X, Lu XG, Zhan LB, Qi X, Liang LN, et al (2011) The effects of the Chinese medicine ZiBuPiYin recipe on the hippocampus in a rat model of diabetes-associated cognitive decline: a proteomic analysis. Diabetologia 54: 1888–1899. 21509442 10.1007/s00125-011-2147-z

[9] Zhan LB, Sui H, Lu XG, Sun CK, Zhang J, et al (2008) Effects of ZibuPiyin recipe on SNK-SPAR pathway in neuron injury induced by glutamate. Chin J Integr Med 14: 117–122. 18679602 10.1007/s11655-008-0117-1

[10] Zhan LB, Niu XP, Sui H, Gong XY (2009) Protective effect of spleen- yin- nourishing recipe on amyloid beta-peptide-induced damage of primarily cultured rat hippocampal neurons and its mechanism. Zhong Xi Yi Jie He Xue Bao 7: 242–248. 19284954 10.3736/jcim20090309

[11] Dinel A-L, Andre' C, Aubert A, Ferreira G, Laye' S, et al (2011) Cognitive and Emotional Alterations Are Related to Hippocampal Inflammation in a MouseModel of Metabolic Syndrome. PLoS ONE 6: e24325. 21949705 10.1371/journal.pone.0024325 PMC3174932

[12] Terry AV Jr (2009) Chapter 13 Spatial Navigation (Water Maze) Tasks. In: Buccafusco JJ, editor. Methods of Behavior Analysis in Neuroscience. 2nd edition. Boca Raton (FL): CRC Press.

[13] Valentinuzzi VS, Menna-Barreto L, Xavier GF (2004) Effect of Circadian Phase on Performance of Rats in the Morris Water Maze Task. Journal of biological rhythms 19: 312–324. 15245650 10.1177/0748730404265688

[14] Murphy GG (2013) Spatial Learning and Memory—What's TLE Got To Do With It? Epilepsy Currents 13: 26–29. 23447735 10.5698/1535-7511-13.1.26 PMC3577082

[15] Penzes P, Cahill ME, Jones KA, VanLeeuwen JE, Woolfrey KM (2011) Dendritic spine pathology in neuropsychiatric disorders. Nat Neurosci 14: 285–293. 21346746 10.1038/nn.2741 PMC3530413

[16] Kulkarni VA, Firestein BL (2012) The dendritic tree and brain disorders. Molecular and Cellular Neuroscience 50: 10–20. 22465229 10.1016/j.mcn.2012.03.005

[17] González-Ramírez MM, Velázquez-Zamora DA, Olvera-Cortés ME, González-Burgos I (2014) Changes in the plastic properties of hippocampal dendritic spines underlie the attenuation of place learning in healthy aged rats. Neurobiology of Learning and Memory 109: 94–103. 24316372 10.1016/j.nlm.2013.11.017

[18] Irvine EE, Drinkwater L, Radwanska K, Qassab HA, Smith MA, et al (2011) Insulin receptor substrate 2 is a negative regulator of memory formation. Learn Memory 18: 375–383. 10.1101/lm.2111311 PMC4371579 21597043

[19] Keith D, Husseini AE (2008) Excitation control: Balancing PSD-95 function at the synapse. Front Mol Neurosci 1: 4. 18946537 10.3389/neuro.02.004.2008 PMC2526002

[20] Rønn SG, Billestrup N, Mandrup-Poulsen T (2007) Diabetes and suppressors of cytokine signaling proteins. Diabetes 56: 541–548. 17259405 10.2337/db06-1068

[21] Starr R, Willson TA, Viney EM, Murray LJ, Rayner JR, et al (1997) A family of cytokine-inducible inhibitors of signaling. Nature 387: 917–921. 9202125 10.1038/43206

[22] Clodfelder-Miller B, De Sarno P, Zmijewska AA, Song L, Jope RS (2005) Physiological and Pathological Changes in Glucose Regulate Brain Akt and Glycogen Synthase Kinase-3. The Journal of Biological Chemistry 280: 39723–39731. 16179343 10.1074/jbc.M508824200 PMC1361688

[23] Dehmelt L, Halpain S (2005) The MAP2/Tau family of microtubule-associated proteins. Genome Biology 6: 204. 15642108 10.1186/gb-2004-6-1-204 PMC549057

[24] Stranahan AM, Arumugam TV, Cutler RG, Lee K, Egan JM, et al (2008) Diabetes impairs hippocampal function through glucocorticoid-mediated effects on new and mature neurons. Nature Neuroscience 11: 309–317. 18278039 10.1038/nn2055 PMC2927988

[25] Oomura Y, Aou S, Fukunaga K (2010) Prandial increase of leptin in the brain activates spatial learning and memory. Pathophysiology 17: 119–127. 19608390 10.1016/j.pathophys.2009.04.004

[26] Jones TS, Knafo S (2012) Spines, Plasticity, and Cognition in Alzheimer's Model Mice. Neural Plasticity 2012: 1–10. 10.1155/2012/319836 PMC3238410 22203915

[27] Bhatt DH, Zhang SX, Gan WB (2009) Dendritic spine dynamics. Annual Review of Physiology 71: 261–282. 10.1146/annurev.physiol.010908.163140 19575680

[28] Hamilton GF, Whitcher LT, Klintsova AY (2010) Postnatal binge-like alcohol exposure decreases dendritic complexity while increasing the density of mature spines in mPFC layer II/III pyramidal neurons. Synapse 64: 127–135. 19771589 10.1002/syn.20711 PMC2789893

[29] Hains AB, Vu MAT, Maciejewski PK, van Dyck CH, Gottron M, et al (2009) Inhibition of protein kinase C signaling protects prefrontal cortex dendritic spines and cognition from the effects of chronic stress. Proc Natl Acad Sci U S A 106: 17957–17962. 19805148 10.1073/pnas.0908563106 PMC2742406

[30] Li XL, Lv YL, Yu S, Zhao HH, Yao LJ (2012) The effect of cadmium on Aβ levels in APP/PS1 transgenic mice. Exp Ther Med 4: 125–130. 23060935 10.3892/etm.2012.562 PMC3460286

[31] Perez SE, Nadeem M, Sadleir KR, Matras J, Kelley CM, et al (2012) Dimebon alters hippocampal amyloid pathology in 3xTg-AD mice. Int J Physiol Pathophysiol Pharmacol 4: 115–127. 23071869 PMC3466490

[32] Talbot K, Wang HY, Kazi H, Han LY, Bakshi KP, et al (2012) Demonstrated brain insulin resistance in Alzheimer's disease patients is associated with IGF-1 resistance, IRS-1 dysregulation, and cognitive decline. J Clin Invest 122: 1316–1338. 22476197 10.1172/JCI59903 PMC3314463

[33] Tezapsidis N, Johnston JM, Smith MA, Ashford JW, Casadesus G, et al (2009) Leptin: A Novel Therapeutic Strategy for Alzheimer's Disease. Journal of Alzheimer's Disease 16: 731–740. 10.3233/JAD-2009-1021 PMC2908903 19387109

[34] Morrison CD (2009) Leptin signaling in brain: A link between nutrition and cognition? BBA-Molecular Basis of Disease 1792: 401–408. 19130879 10.1016/j.bbadis.2008.12.004 PMC2670357

[35] Lee CC, Huang CC, Hsu KS (2011) Insulin promotes dendritic spine and synapse formation by the PI3K/Akt/mTOR and Rac1 signaling pathways. Neuropharmacology 61: 867–879. 21683721 10.1016/j.neuropharm.2011.06.003

[36] Zhang J, Matheny MK, Tumer N, Mitchell MK, Scarpace PJ (2007) Leptin antagonist reveals that the normalization of caloric intake and the thermic effect of food after high-fat feeding are leptin dependent. American Journal of Physiology-Regulatory, Integrative and Comparative Physiology 292: R868–R874. 10.1152/ajpregu.00213.2006 17023670

[37] Guo Z, Jiang H, Xu X, Duan W, Mark P Mattson (2008) Leptin-mediated cell survival signaling in hippocampal neurons mediated by JAK STAT3 and mitochondrial stabilization. The Journal of Biological Chemistry 283: 1754–1763. 17993459 10.1074/jbc.M703753200

[38] Belfiore A, Frasca F, Pandini G, Sciacca L, Vigneri R (2009) Insulin receptor isoforms and insulin receptor/insulin-like growth factor receptor hybrids in physiology and disease. Endocrine Reviews 30: 586–623. 19752219 10.1210/er.2008-0047

[39] Chiu SL, Cline HT (2010) Insulin receptor signaling in the development of neuronal structure and function. Neural Development 5: 7–24. 20230616 10.1186/1749-8104-5-7 PMC2843688

[40] Tello PS, Matamoros AO, Arias C (2011) GSK3 Function in the Brain during Development, Neuronal Plasticity, and Neurodegeneration. International Journal of Alzheimer's Disease 2011: 1–12. 10.4061/2011/189728 PMC3109514 21660241

[41] Hooper C, Killick R, Lovestone S (2008) The GSK3 hypothesis of Alzheimer's disease. Journal of Neurochemistry 104: 1433–1439. 18088381 10.1111/j.1471-4159.2007.05194.x PMC3073119

[42] Zhou FQ, Snider WD (2005) GSK-3β and microtubule assembly in axons. Science 308: 211–214. 15825222 10.1126/science.1110301

[43] Hur EM, Zhou FQ (2010) GSK3 signalling in neural development. Nature Reviews Neuroscience 11: 539–551. 20648061 10.1038/nrn2870 PMC3533361

[44] Shen H, Shao M, Cho KW, Wang S, Chen Z, et al (2012) Herbal constituent sequoyitol improves hyperglycemia and glucose intolerance by targeting hepatocytes, adipocytes, and β-cells. Am J Physiol Endocrinol Metab 302: E932–E940. 22297305 10.1152/ajpendo.00479.2011 PMC3330724

[45] Yin J, Zuberi A, Gao Z, Liu D, Liu Z, et al (2009) Shilianhua extract inhibits GSK-3β and promotes glucose metabolism. Am J Physiol Endocrinol Metab 296: E1275–E1280. 19351808 10.1152/ajpendo.00092.2009 PMC2692393

