# Supplementary figures and images for: ZiBuPiYin Recipe Protects db/db Mice from Diabetes-Associated Cognitive Decline through Improving Multiple Pathological Changes
Source: PLoS One. 2014 Mar 10;9(3):e91680. doi: 10.1371/journal.pone.0091680 (PMC3948870; doi:10.1371/journal.pone.0091680)

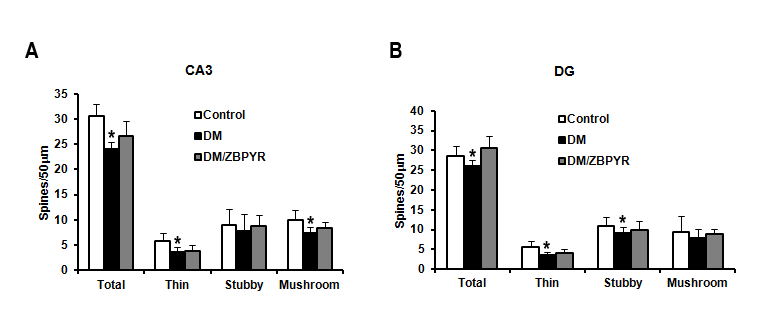

Supplement: Figure S1 — Effects of ZBPYR on dendritic spines in CA3 and DG of hippocampus. (A) In the CA3 of hippocampus in DM mice, the total dendritic spine density, thin-type spine density and mushroom-type spine density were significantly decreased. (B) In the DG of hippocampus in DM mice, the total dendritic spine density and the density of thin- and stubby-type spine were significantly decreased. Values are means ± S.D. from 3 mice in each group. *p<0.05 compared to control. (TIF) [file pone.0091680.s001.tif]

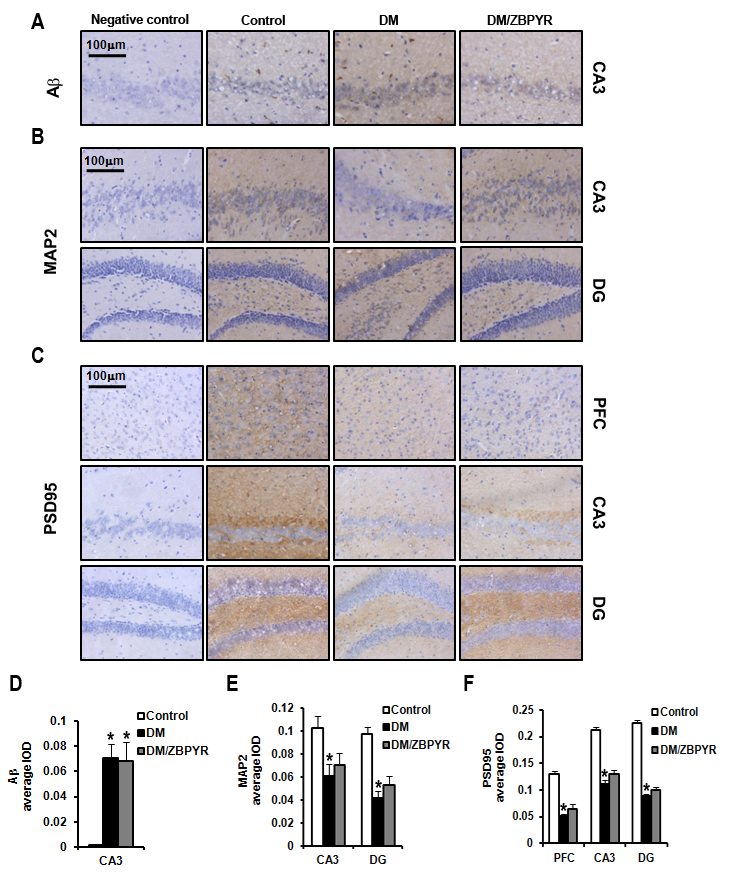

Supplement: Figure S2 — Effects of ZBPYR on Aβ1-42 deposition and the expression of neurostructural proteins in different brain region. (A) Aβ1-42 deposition in the hippocampal CA3. (B) MAP2 expression in the hippocampal CA3 and DG. (C) The expression of PSD95 in the PFC, hippocampal CA3 and DG. (D–F) The average IOD of Aβ1-42 (D), MAP2 (E) and PSD95 (F) are shown as bar graphs. Values are means ± S.D. from 3 mice in each group. *p<0.05 compared to control. (TIF) [file pone.0091680.s002.tif]
